# Renal detection of *Plasmodium falciparum*, *Plasmodium vivax* and *Plasmodium knowlesi* in malaria associated acute kidney injury: a retrospective case–control study

**DOI:** 10.1186/s13104-020-4900-1

**Published:** 2020-01-20

**Authors:** Charandeep Kaur, Atreyi Pramanik, Kalpana Kumari, Rajendra Mandage, Amit Kumar Dinda, Jhuma Sankar, Arvind Bagga, Sanjay Kumar Agarwal, Aditi Sinha, Geetika Singh, Pragyan Acharya

**Affiliations:** 1 Department of Biochemistry, All India Institute of Medical Sciences, New Delhi, India; 2 Department of Pathology, All India Institute of Medical Sciences, New Delhi, India; 3 Department of Pediatrics, All India Institute of Medical Sciences, New Delhi, India; 4 Department of Nephrology, All India Institute of Medical Sciences, New Delhi, India

**Keywords:** Acute kidney injury (AKI), *Plasmodium*, *falciparum*, *vivax*, *knowlesi*, Complicated malaria, Severe malaria, Renal tissue

## Abstract

**Objective:**

Acute kidney injury (AKI) is a frequent presentation in malaria infections. Several cases of AKI that are accompanied by clinical symptoms of malaria infection, such as fever, nausea, respiratory distress, and anemia remain undiagnosed due to challenges in accurate diagnosis using peripheral blood microscopy and rapid diagnostic tests that are currently used in clinical settings. This is particularly true for *P. vivax* and *P. knowlesi* infections. As a result, these patients are not able to receive anti-malarial therapy in a timely manner. The objective of the present study was to investigate if patients presenting with AKI harbored any of the five human *Plasmodium* species (*P. falciparum*, *P. vivax*, *P. knowlesi*, *P. malariae*, and *P. ovale*) within their renal tissues.

**Results:**

We found that renal biopsies from malaria associated AKI patients harbor the human malaria parasites *P. falciparum*, *P. vivax* and *P. knowlesi* as mono- and mixed species infections. Presence of microvascular injury in a majority of the malaria associated AKI cases suggested vascular involvement of *P. vivax* and *P. knowlesi*. This research note also highlights *P. knowlesi* as an emerging pathogen in the Indian subcontinent.

## Introduction

Human malaria is caused by five different species of *Plasmodium*–*P. falciparum*, *P. vivax*, *P. knowlesi*, *P. malariae* and *P. ovale*. Acute kidney injury (AKI), is a frequent presentation in severe malaria which is associated with mortality [1–5]. Overall, AKI has been shown to have a global prevalence in about 20–50% hospitalized malaria cases [6, 7]. While *P. falciparum* is found all over the tropical world, *P. vivax* is highly prevalent in South America, India and South East (S. E.) Asia, and *P. knowlesi* has been shown to cause malaria predominantly in S.E. Asia [8–11] with a single report from the Andaman and Nicobar Islands of India [12]. In many malaria cases in endemic areas severe malaria symptoms such as AKI manifest, but malaria diagnosis by microscopy or RDT is made only in a proportion of these cases, and the rest remain as occult malaria infections [13–16]. An earlier study from our center, based on which this study has been built, had reported the presence of peripheral *P. vivax* infection in patients with AKI [17]. However, the presence of the malaria parasites within the renal tissue of AKI patients was not demonstrated. As a result, in the earlier study many AKI patients with malaria-like symptoms and without a clear malaria diagnosis were excluded. We hypothesized that in spite of negative peripheral smear and RDT, many of the AKI cases presenting with malaria- like symptoms could be cases of occult malaria infections which harbor parasites localized to the renal tissue. The objectives of the present study were– (a) To investigate if AKI cases with malaria-like symptoms but without a clear diagnosis harbored the *Plasmodium* species within their renal tissue and, (b) To investigate the presence of all five species of *Plasmodium* known to infect humans (*P. falciparum*, *P. vivax*, *P. knowlesi*, *P. malariae*, and *P. ovale*) in malaria AKI cases since most diagnostic methods are suitable for the detection only of *P. falciparum* and *P. vivax*. We report our observations here.

## Main text

### Methods

#### Study design

This study was a retrospective case–control analysis carried out as collaboration of the Department of Biochemistry with the Departments of Pathology, Pediatrics and Nephrology at All India Institute of Medical Sciences (AIIMS), New Delhi, India. The ethics committee of the All India Institute of Medical Sciences, New Delhi has approved this study. The overall study design is presented as a flowchart in Additional file 1: Figure S1.

#### Renal tissue samples

Archival formalin-fixed paraffin-embedded (FFPE) renal tissue blocks from 2011 to 2018 were retrieved from the Department of Pathology, AIIMS, New Delhi, India. Geographical distributions of patients are presented in Additional file 1: Figure S2.

- Cases: AKI patients presented with abdominal pain, fever and low urine output with suspected or confirmed malaria diagnosis. Both peripheral smear malaria positive cases with confirmed malaria diagnosis, as well as peripheral smear malaria negative cases with malaria-like symptoms have been included as cases.
- Controls: Renal biopsies from AKI cases showing acute tubular injury or necrosis (ATN) and/or acute cortical necrosis (ACN) without clinical, laboratory, or histopathological evidence of malaria. Details of malaria AKI cases are presented in Table 1 and of controls are presented in Table 2.

**Table 1:** Summary of clinical presentation of AKI in patients with malaria infection with PCR diagnosis of the five *Plasmodium* species infecting humans

Sample Id	Age (years)	Clinical presentation	Laboratory investigations	Blood smear evidence	Histology	PCR diagnosis of renal tissue DNA
AIIMSK8299	23	Sudden onset placental abruption	Sudden rise of creatinine (4.2 mg/dl)	Nil	Patchy ACN	*Pv *+ *Pk*
AIIMSK6691	22	Fever and edema 1 month, hematuria, hypertensive retinopathy,CM	Low C3 level, LDH elevated, urine: RBCs full field	Nil	ATI and subacute TMA	*Pf *+ *Pk *+ *Pv*
AIIMSK1056	8	Fever, oliguria,CM	Creatinine-3.6 mg/dl, thrombocytopenia	Nil	ACN (70–75%), viable single artery-fibrin thrombus (TMA)	*Pk *+ *Pv*
AIIMSK3873	30	Fever, oliguria-3 months, CM	Creatinine-5.9 mg/dl, proteinuria (2 +), HD dependent	Nil	Patchy cortical necrosis (scarring phase), acute interstitial nephritis, vessels -UR	*Pv *+ *Pk *+ *Pf*
AIIMSK0447	37	Hematuria	Case of TMA on PLEX, on rituximab	Nil	Chronic TMA	*Pv* + *Pk* + *Pf*
AIIMSK4424	17	Fever, right-sided hemiplegia, oliguria-15 days,CM	Deranged RFT, LDH elevated	Nil	ATN, interstitial inflammation, IFTA (20%)	*Pv *+ *Pk *+* Pf*
AIIMSK7559	20	Complicated malaria (one and half months back), Sepsis, anuria, myocarditis	Creatinine-4.1, thrombocytopenia, proteinuria (2 +), HD dependent	*Pv* trophozoites	ACN (60%)	*Pv*
AIIMSK2788	22	Fever, oliguria, melena, jaundice, cardiac dysfunction	Creatinine-9.4, thrombocytopenia, proteinuria (1 +)	*Pv* trophozoites	Patchy ACN	*Pv*
AIIMSK9006	10	Fever and oliguria-6 days, epistaxis, melena, splenomegaly	Creatinine-6.2 mg/dl, thrombocytopenia, urine: proteinuria (4 +), 8–10 RBC/hpf, raised LDH	*Pv* trophozoites	ATN, TMA	*Pv*
AIIMS0066	21	Fever-10 days, oliguria-2 days, splenomegaly	Creatinine-6.5 mg/dl, thrombocytopenia, proteinuria (1 +), raised LDH	Nil	ACN secondary to graft artery thrombosis	*Pk*
AIIMSK7006	30	Fever, oliguria	Creatinine-7.2 mg/dl, thrombocytopenia, proteinuria (2 +), raised LDH, HD-dependent,	*Pv* trophozoites	Multifocal cortical necrosis with scarring, ATI, chronic TMA	*Pv*
AIIMSK0339	50	Fever-10 days, oliguria-2 days, splenomegaly	Creatinine-8.2 mg/dl, Underwent rectopexy for rectal prolapse; POD3- hematuria, proteinuria, on HD	*Pv* trophozoites	ACN, TMA	*Pv*

*ACN* acute cortical necrosis, *ATI* acute tubular injury, *TMA* thrombotic microangiopathy, *AIN* Acute Interstitial Nephrosis, *IFTA* Interstitial Fibrosis Tubular Atrophy, *RFT* Renal Function Test, *HD* Hemodialysis, *CM* complicated malaria, *LDH* lactate dehydrogenase, *Pf Plasmodium falciparum*, *Pv Plasmodium vivax*, *Pk Plasmodium knowlesi*. *Plasmodium ovale* and *Plasmodium malariae* were not detected in these samples

**Table 2:** Clinical details of the control group

Sample ID	Age (years)	Specimen	Clinical features and laboratory investigations	Histology	PCR findings
AIIMSK3235	20	Nephrectomy	Case of LN, post-biopsy abdominal distension and fall of hemoglobin	ACN, LN class-IV + V	No evidence of *Plasmodium* species
AIIMSK3417	15	Biopsy	Renal allograft recipient; oliguria and rising creatinine	Diffuse ACN	
AIIMSK7167	6	Biopsy	Thrombocytopenia, anemia, hyperkalemia, deranged RFT, LDH elevated urine: proteinuria (2 +), 10–15 RBC/hpf	ACN (45–50%), viable area-ATI, Arteries and arterioles unremarkable	
AIIMSK6701	11	Biopsy	Creatinine-7.9 mg/dl, thrombocytopenia, urine: proteinuria (2 +), 15–20 RBC/hpf, raised LDH	Resolving ACN, AIN, mesangiolysis, arterioles-vacuolization and endothelial swelling	
AIIMSK6683	12	Biopsy	Creatinine-10.3 mg/dl, thrombocytopenia, urine: proteinuria (1 +), 8–10 RBC/hpf, raised LDH	Resolving ATN	
AIIMSK7384	19	Biopsy	Creatinine-4 mg/dl, thrombocytopenia, urine: proteinuria (2 +), 8–10 RBC/hpf, raised LDH	Focal ACN, TMA, blackish-brown pigments glomerulus, tubules and interstitium	
AIIMSK3326	18	Biopsy	Creatinine-9.8 mg/dl, thrombocytopenia, urine: proteinuria (4 +), 15–20 RBC/hpf, raised LDH	Resolving ATN with focal mesangiolysis	
AIIMSK6641	9	Biopsy	Creatinine-6.5 mg/dl, thrombocytopenia, proteinuria (1 +), raised LDH	Resolving ATN with AIN. Arteries and arterioles-endothelial swelling	

*LN* lupus nephritis, *LSCS* lower segment caesarean section, *PS* peripheral smear

The individuals who performed the experiments were blinded to the identity of the controls and cases until the PCR outcomes had been obtained and documented.

Initial evaluation of patients included urine analysis, ultrasound, complete blood counts and measurements of creatinine, urea, electrolytes, pH and bicarbonate. Renal biopsies were processed for light microscopy by standard techniques. Diagnosis of malaria was based on peripheral blood smears.

#### Histological examination

Histological sections of all FFPE tissues were stained with Giemsa, hematoxylin, periodic acid schiff (PAS) and Jones methenamine silver stains [18]. The glomeruli, tubules, interstitium, and blood vessels were examined in all the cores. To confirm that the crystals observed in the sections were hemozoin, a saturated solution of picric acid in ethanol was used (malarial bleach) [19].

#### Detection of malaria parasites

- Clinical diagnosis of malaria was based on microscopic detection of *Plasmodium* species in the peripheral blood smear (thick and thin smears stained by Giemsa). Species identification was done on thin film microscopy (Nikon Ni-E). In the cases where peripheral blood smear did not yield any parasites, diagnosis was carried out by physicians based on clinical presentation.
- For detection of malaria parasite from renal tissue, DNA was extracted using the Qiagen QiAmp DNA FFPE tissue kit as per the manufacturer’s protocol. PCR amplification of extracted DNA for all the five *Plasmodium* species (*P. falciparum, P. vivax, P. knowlesi, P. malariae* and *P. ovale*) was carried out. The primers used for PCR amplification are listed in Additional file 1: Table S1. The PCR products were subjected to Sanger sequencing (Bencos Research Solutions Pvt. Ltd.) in order to confirm the species identity (Additional file 1: Table S2). The *P. knowlesi* primers were designed to target Pkr140 gene, which is present in 7 copies distributed across 6 different chromosomes in the *P. knowlesi* genome [20]. These primers designed by Lucchi et al., detect *P. knowlesi* specific DNA segments which are not present in the other human *Plasmodium* species and therefore, prevent cross reactivity with either *P. vivax* or *P. falciparum*. At least 150 ng of DNA was used in a 20 µl PCR reaction.

#### Generation of the map of India

Indian map shapefile (.shp) was downloaded from http://www.indianremotesensing.com/2017/01/Download-India-shapefile-with-kashmir.html. Then, the map was visualized and saved using map shaper tool https://mapshaper.org/. The zoomed in image of Delhi was by using R package “rgdal” only. The final composite map was generated using Microsoft Powerpoint.

### Results

#### PCR evidence of *Plasmodium* Species in FFPE renal biopsies from AKI cases

All the 12 cases and 8 controls were subjected to PCR analysis for all the 5 *Plasmodium* species (*P. falciparum*, *P. vivax*, *P. knowlesi*, *P. malariae* and *P. ovale*). Among the 12 cases, 5 cases were found to have *P. vivax*, 1 had *P. knowlesi*, 2 had mixed infections of *P. vivax* and *P. knowlesi*, and 4 had mixed infections consisting of three parasites *P. vivax*, *P. falciparum*, and *P. knowlesi* (Table 1). Overall, of 12 cases with malaria associated AKI, the renal tissue of 11 had *P. vivax*, 7 had *P. knowlesi* and, 4 had *P. falciparum* infection. Interestingly, of the 7 cases of malarial AKI having *P. knowlesi* by PCR, all lacked peripheral smear evidence of the parasite (Table 1) but 4 presented as CM, 1 had placental abruption with AKI, and 1 presented with TMA and required plasmapheresis (Table 1). The *P. knowlesi* PCR products were Sanger sequenced followed by NCBI BLAST in order to confirm species identity (Additional file 1: Table S2).

None of the control FFPE renal biopsies contained parasitic DNA by PCR analysis (Table 2).

The origin of all malaria associated AKI cases could be mapped to Haryana, Uttarakhand, Delhi, Bihar and Uttar Pradesh in India (Additional file 1. Figure S2).

#### Presentation and histopathological analysis of AKI cases

**Fig. 1:**
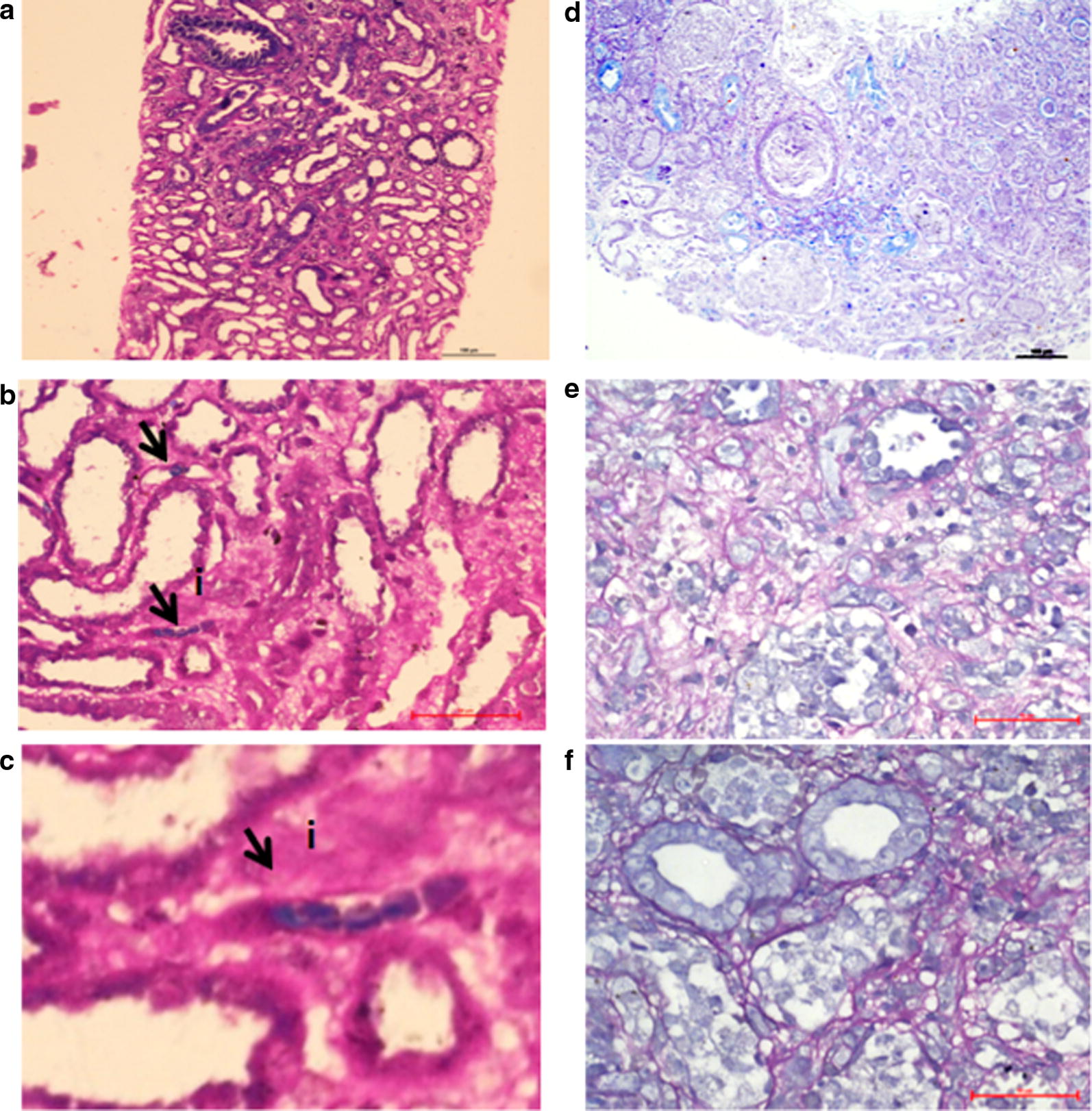
Photomicrograph depicting differences in Giemsa stained renal tissue from *Plasmodium* PCR positive sample for Pv and Pk positive renal core (**a**–**c**) vs. *Plasmodium* PCR negative sample (**d**–**f**). Giemsa stain highlights ring stage parasites (**a**, 10×, **b**, 40×, black arrows indicate parasites and **c**, inset: expanded image of parasites indicated by black arrows in **b**) within renal peritubular capillaries consistent with the presence of the malaria parasite. *Plasmodium* PCR negative renal biopsy did not demonstrate similar structures in the photomicrograph (**d**, 10×; **e**, 20× and **f**, 40×)

In the present study, 12 FFPE samples (Table 1) with malaria associated AKI and 8 control samples (AKI with non-malarial etiology) (Table 2) collected from 2011 to 2018 were included. Of the 12 cases, 9 presented with fever and associated oliguria, 2 presented with fever and associated hematuria; 1 as hematuria alone; 1 presented as AKI about one and half month after diagnosis as complicated *P. vivax* malaria; and 1 had presented with placental abruption associated with AKI (Table 1; column 3). The various histological diagnoses in these cases were acute cortical necrosis (ACN), acute tubular injury/necrosis (ATI/ATN), thrombotic microangiopathy (TMA), and interstitial fibrosis and tubular atrophy (IFTA). All the 12 cases had evidence of cortical necrosis, TMA, or mesangiolysis, indicating vascular involvement (Table 1 and Fig. 1).

The peripheral blood smears of 12 malaria AKI cases revealed *P. vivax* infections in 5 samples; and no evidence of peripheral blood parasitemia in 7 samples (Table 1, Column 5). Of these 7 samples, 4 were clinically diagnosed as complicated malaria (CM), 1 presented with hematuria and 1 with placental abruption (Table 1). None of the control samples had any symptoms resembling malaria or any evidence of malaria from peripheral blood smear. They presented with ACN and TMA associated with non-malaria cases as described in Table 2.

### Discussion

The association of AKI with peripheral blood *P. falciparum*, *P. vivax and P. knowlesi* infection has been reported by several groups [1, 2, 21–23]. We demonstrate direct tissue presence of the parasite derived DNA, hemozoin as well as infected RBCs for these species. Interestingly, of the 12 cases, 7 had no evidence of the parasite from microscopic examination of peripheral blood smears. Therefore, our study corroborates the limitations of microscopy in the detection of *P. vivax* and *P. knowlesi,* that have been clearly demonstrated in large scale studies in S. E. Asia [13, 14] Our study also suggests that *P. knowlesi*, which is thought to be predominantly a S. E. Asian parasite, is now also emerging in the Indian mainland.

In addition to PCR evidence of the presence of parasite DNA, we found specific staining for iron containing hemozoin, which was completely absent in all the control samples. Hemozoin pigment is known to be produced only by metabolically active, replicating parasites [24]. When in circulation, it is rapidly phagocytosed by macrophages and dendritic cells and therefore, has a short circulating half life [25]. Hemozoin pigment in renal tissue has been demonstrated in instances of *P*. *falciparum* associated AKI [6]. Therefore, presence of hemozoin staining specifically in renal tubules and interstitium is suggestive of the presence of replicating parasites at the site, rather than non-specific accumulation of hemozoin [26].

The epidemiological importance of our study is that currently in India, and several other neighboring regions *P. knowlesi* is not included in preliminary diagnoses based on the belief that it does not exist in these regions [27]. We demonstrate that this is not the case in mainland India and along with one previous report on *P. knowlesi* from the Andaman and Nicobar islands, we show that *P. knowlesi* is present in the Indian subcontinent [12]. This is not surprising in present times since there is tremendous amount of mobility within populations and across distant geographical regions. Therefore, pathogens too have the ability to travel through their hosts and reservoirs, across political borders and this has been shown to happen to several pathogens including malaria. In fact resurgence of malaria in several places is attributed to international travel by their human hosts [28].

Secondly, our study shows that cases of AKI associated with malaria like symptoms but unconfirmed malaria diagnosis by microscopy or RDT, such as seen in the cases reported in Table 1, may be cases of occult *P. vivax* or *P. knowlesi* infections. Therefore, we recommend that *P. knowlesi* must be included in preliminary screening of patients suspected to have malaria in India, as well as in all those regions where malaria is endemic and where the vectors and reservoirs of *P. knowlesi* have a home.

### Strengths of the study

1. This study highlights *P. knowlesi* as an emerging pathogen in the Indian subcontinent.
2. This study shows the presence of *P. falciparum*, *P. vivax* and *P. knowlesi* within the renal tissue even in individuals with negative peripheral blood smear results thereby emphasizing the importance of developing new molecular detection methods for malaria.
3. The presence of *P. knowlesi* in mainland India clearly reveals the importance of screening patients for all malaria species rather than the most prevalent ones since the less prevalent species may escape elimination efforts by escaping diagnosis.

## Limitations

1. This study is not an in-depth mechanistic or epidemiological prevalence analysis and therefore, cannot provide a deeper insight into molecular aspects of host–pathogen interactions during malarial AKI or a wider perspective on the incidence of *P. vivax* or *P. knowlesi* cases in malarial AKI. However, it shows that *P. vivax* and *P. knowlesi* are present within renal tissue samples of AKI patient and highlights the emergence of *P. knowlesi* in mainland India.
2. Many of the FFPE renal tissue samples from the study are peripheral smear negative for malaria but positive by PCR analysis. Since this is a retrospective study of the archived tissue samples, blood samples from the samples patients are not available for further analysis.

## Supplementary information

**Additional file 1: Figure S1.** Flow chart representing study design. **Figure S2.** Geographical distribution of patients harboring malaria associated AKI. Data was not available for 9 samples (3 controls, 6 cases). **Table S1.** Oligonucleotide sequences for *Plasmodium* species used for PCR amplification using the standardized conditions. **Table S2.** BLAST results of Sanger sequenced PCR products of *Plasmodium knowlesi.*

## Data Availability

All datasets supporting the conclusions of this article is included within the article and as supplementary files.

## Abbreviations

AKI: acute kidney injury
*P. falciparum*: *Plasmodium falciparum*
*P. vivax*: *Plasmodium vivax*
*P. knowlesi*: *Plasmodium knowlesi*
S. E. Asia: South East Asia
RDT: rapid diagnostic test
FFPE: formalin-fixed paraffin-embedded
ATN: acute tubular injury or necrosis
ACN: acute cortical necrosis
PAS: periodic Acid schiff
TMA: thrombotic microangiopathy
IFTA: interstitial fibrosis and tubular atrophy
CM: complicated malaria
