# Supplementary material for: Renal detection of Plasmodium falciparum, Plasmodium vivax and Plasmodium knowlesi in malaria associated acute kidney injury: a retrospective case–control study
Source: BMC Res Notes. 2020 Jan 20;13:37. doi: 10.1186/s13104-020-4900-1 (PMC6971858; doi:10.1186/s13104-020-4900-1)
Supplement: Supplementary file 1 — Additional file 1: Figure S1. Flow chart representing study design. Figure S2. Geographical distribution of patients harboring malaria associated AKI. Data was not available for 9 samples (3 controls, 6 cases). Table S1. Oligonucleotide sequences for Plasmodium species used for PCR amplification using the standardized conditions. Table S2. BLAST results of Sanger sequenced PCR products of Plasmodium knowlesi. [file 13104_2020_4900_MOESM1_ESM.pdf]

Additional file 1

Renal detection of Plasmodium falciparum, Plasmodium vivax and Plasmodium knowlesi in malaria associated acute kidney injury: a retrospective case–control study

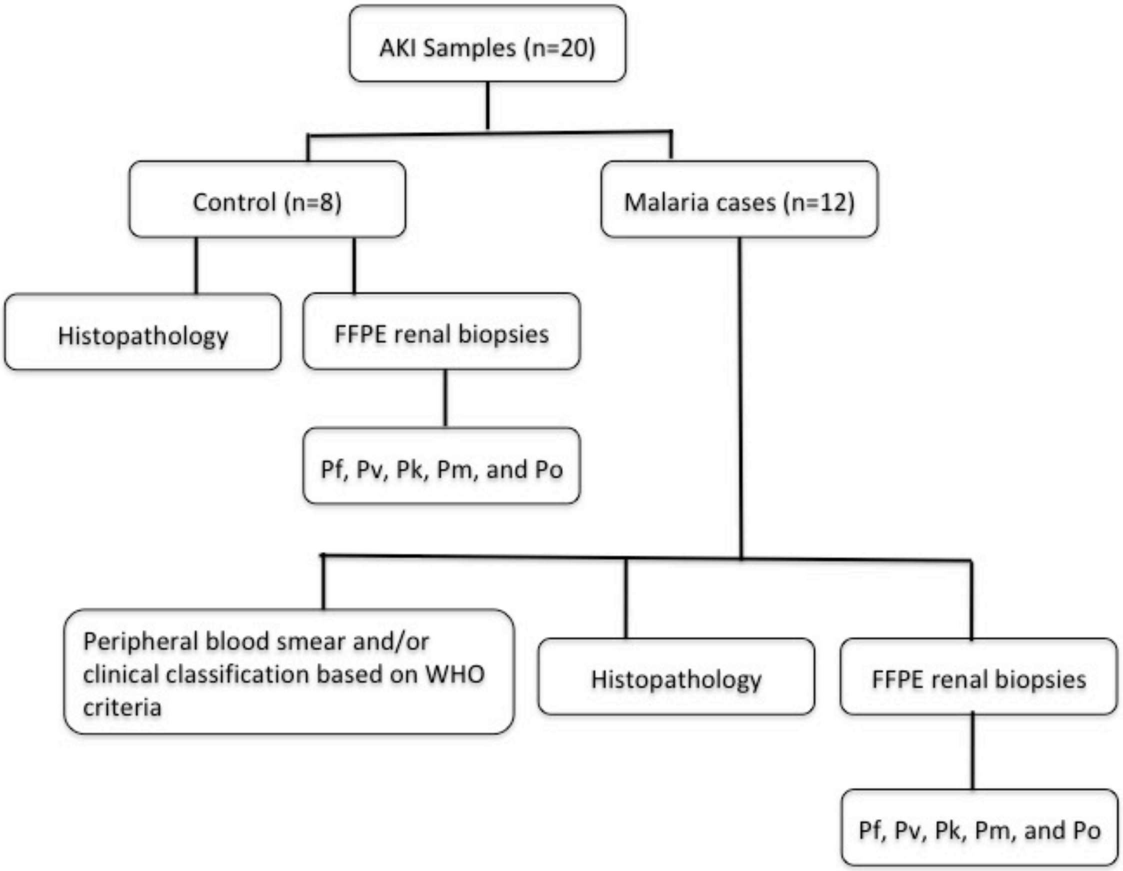

Figure S1. Flow chart representing study design. This was a retrospective analysis wherein archival FFPE renal biopsies were taken from AKI patients with malaria or without malaria. They were subjected to DNA extraction and PCR for all the 5 human malaria species (Pf= *P. falciparum*, Pv= *P. vivax*, Pk= *P. knowlesi*, Pm= *P. malariae*, Po= *P. ovale*). Histopathological analysis and existing peripheral smear data were analyzed with PCR outcomes.

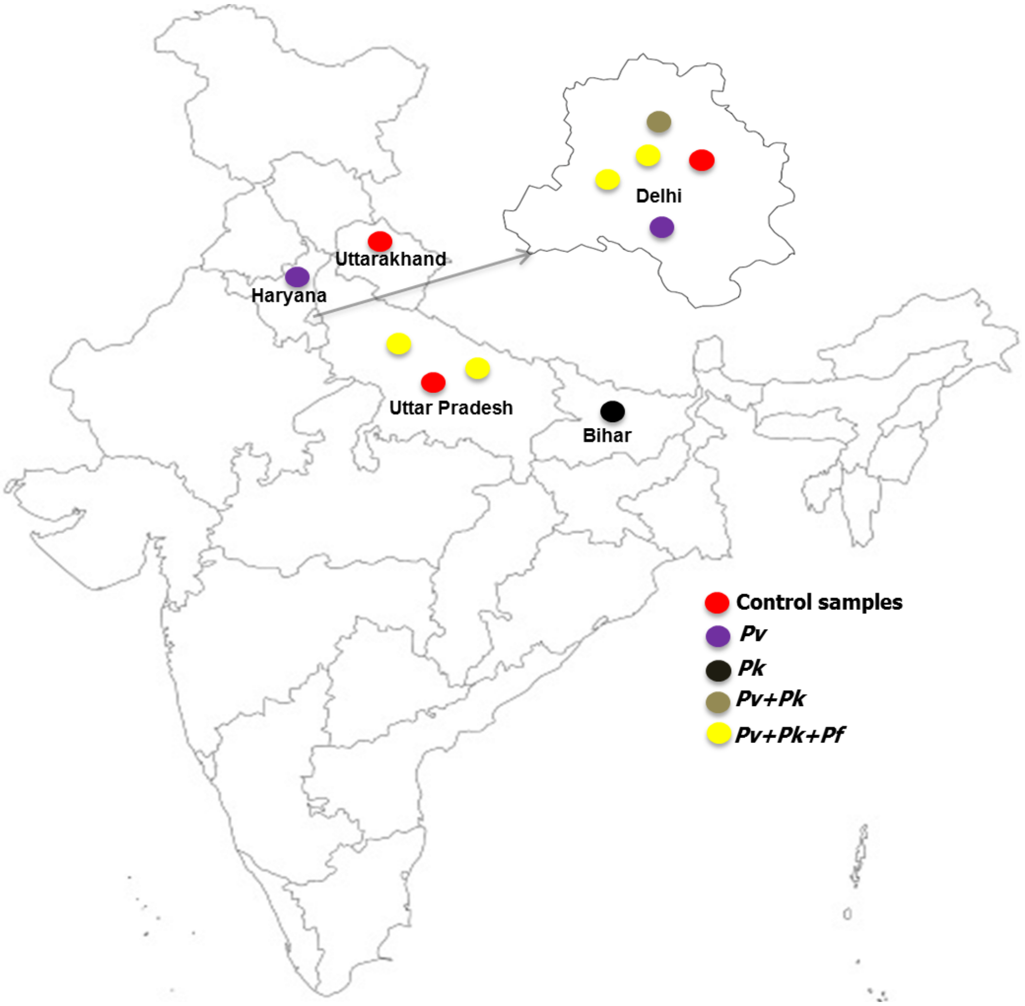

Figure S2. Geographical distribution of patients harboring malaria associated AKI. Data was not available for 9 samples (3 controls, 6 cases).

Table S1: Oligonucleotide sequences for P*lasmodium* species used for PCR amplification using the standardized conditions

***Plasmodium* species**	**Forward primer (5'-3')**	**Reverse primer (5'-3')**
Pf [1]	TTAAACTGGTTTGGGAAAACCAAATATATT	ACACAATGAACTCAATCATGACTACCCGTC
Pv[1]	CGCTTCTAGCTTAATCCACATAACTGATAC	ACTTCCAAGCCGAAGCAAAGAAAGTCCTTA
Pm[1]	ATAACATAGTTGTACGTTAAGAATAACCGC	AAAATTCCCATGCATAAAAAATTATACAAA
Po[1]	ATCTCTTTTGCTATTTTTTAGTATTGGAGA	GGAAAAGGACACATTAATTGTATCCTAGTG
Pk[2]	CAGAGATCCGTTCTCATGATTTCCATGG	CTRAACACCTCATGTCGTGGTAG

1. Snounou G, Viriyakosol S, Xin Ping Zhu, et al. High sensitivity of detection of human malaria parasites by the use of nested polymerase chain reaction. Mol Biochem Parasitol **1993**; 61:315–320.

2. Lucchi NW, Poorak M, Oberstaller J, et al. A new single-step PCR assay for the detection of the zoonotic malaria parasite Plasmodium knowlesi. PLoS One **2012**; 7:e31848.

Table S2: BLAST results of Sanger sequenced PCR products of *Plasmodium knowlesi*

Sample Id	Sequence	Sequence Id	E value	Identity
**Sequences for *Plasmodium knowlesi***				
AIIMSK0447_PK_F	CCCCCAAGACCACGCGGGGGCAACGAATGTAACGTGTTAGGAAGAAAACACCTCAGGATGTGCCAGCATCTTTTGACTTTTATTTTGGGGGGCTGCATCTACTACCACGACATGAGGTGTTCAGAGATCCGGTCTCATGATTTCGGTGTCCGTGATG	LT727658.1	2.9	100%
AIIMSK0447_PK_R	ACCTTAAGGAACCAGAGGAGGAGGAACAGCAGAAGAGGGAATTTCCAGCTACTAACAATTGGTGTAGTTCATCATCACCTGTAATGACCAAAATGTCTCCCATGGAAATCATGACAACGGATCTTTGATTTTCAAA	LT727658.1	2 e^-04	93%
AIIMSK1056_PK_F	CCCCCCAGGGTTGTTGTATGAAACAGAACTCTCATATTGTGAGTCGGATGAAAACAACCTCAGGAATGTGCCAGCATCTTTTGACTTTTATTTTGGGGGGCTGCATCTACTACCACGACATGAGGTGTTCAGAGACCGG	LT727652.1	4e^-06	97%
AIIMSK1056_PK_R	CGGGAGAATAGCAAAGCCACAGTTGGATGAGGGTCTTCTCAGGACTAACAATTGGTGTAGTTCATCATCACCTGGAATGACCAAAATGTCTCCCATGGAAATCATCACCCCGGATCTCGGATTTTCAAAT	LT727669.1	0.030	93%
AIIMSK3873_PK_F	CACTCGACGGTGCAAGGGGGGGCGAAGAGAAAACGTAGTGATGAGAATCCCTCGGGTTGTGCCAGCATCTTTTGACTTTTATTTTGGGGGGCTGCATCTACTACCACGACATGAGGTGTTTAGAGATCCGGACTCATGATTTCGATGGTTATCATGACAGCTCTTCTGCTGA	LT727653.1	6e^-05	100
AIIMSK3873_PK_R	CTTACCAAACCGGGGGGACCGGAGGAGGAGGAACAGAAGAAGAGGGACTTTTCAGCCACTAACAATTGGTGTAGTTCATCATCACCTGGAATGACCAAAATGTCTCCCATGGAAATCATCACCCCGGATCTCTGATTTTCAAA	LT727655.1	0.003	100
AIIMSK4424_PK_F	TGTTGGGGTAAAGAAGGAACCTCCAGCAATTTGTTATGTGGCTGAGAAGAACCTCAGGAATGTGCCAGCATCTTTTGACTTTTATTTTGGGGGGCTGCATCTACTACCACGACATGAGGTGTTCAGAGATCCGTTCTCAGGATCCGATGTTGG	LT727652.1	1e^-05	100
AIIMSK4424_PK_R	CTTTGAAAAACATTCCTAGAGGTTCTTTTAAGCCACTAACAATTTGGGGTAGTTCATCATCACCTGGAATGACCAAAATGTCTCCCATGGAAATCATGAGAACGGATCTCTGACCTCCAAAT	LT727658.1	4e^-07	100
AIIMSK6691_PK_F	CCTTCGAGGGGGGCGCGGCCGGGGGCGAAATGAGAACAGTGAGGCGGTAGAGAGAACCTCAGGAATGTGCCAGCATCTTTTGACTTTTATTTTGGGGGGCTGCATCTACTACCACGACATGAGGTGTTTAGAGATCCGTTCTCATGATTTCGAT	LT727658.1	5e^-06	94

	GGTAGTGATGCGATCCCTTCTGTGA			
AIIMSK6691_PK_R	CTAAAGAGGGGGAGACGGCGGCGCGGGCCTGCCGATGCGGGAATTCTCAACCACAACAATCGTCGTTTTTCTTCATCACCTGTAATGACCAAAATGTCTCCCATGGAAATCATCAGACCGGATCTCTGACCTTCAAA	LT727658.1	2e^-04	93
AIIMSK8299_PK_F	CTGTCGTAATAAATAGAAGAACAGAGACAAATGAGCCGGATGAAAAGAATTCAGGAATGTGCCAGCATCTTTTGACTTTTATTTTGGGGGGCTGCATCTACTACCACGACATGAGGTGTTCAGAGATCCGTTCTCATGATTTCCATGCAACTCATGAGATCTCATCTCCGATG	LT727658.1	1e^-07	100
AIIMSK8299_PK_R	ATTTAGGGAAACATTCGTAGAGGGTTTTCTCAGCCACTAACAATTGGTGTAGTTCATCATCACCTGGAATGACCAAAATGTCTCCCATGGAAATCATGAGAACGGATCTCTGATTTTCAAAT	LT727658.1	4e^-07	100
